# Effect of PTH and corticotomy on implant movement under mechanical force

**DOI:** 10.1186/s12903-020-01310-4

**Published:** 2020-11-10

**Authors:** Jiyeon Kim, Heon-Young Kim, Won-Ho Kim, Jin-Woo Kim, Min-Ji Kim

**Affiliations:** 1 School of Medicine, Ewha Womans University, Seoul, South Korea; 2 Department of Oral and Maxillofacial Surgery, School of Medicine, Ewha Womans University, Anyangcheon-ro 1071, Yangcheon-gu, Seoul, 158-710 South Korea; 3 Department of Orthodontics, School of Medicine, Ewha Womans University, Anyangcheon-ro 1071, Yangcheon-gu, Seoul, 158-710 South Korea

**Keywords:** Implant, Bone remodeling, Parathyroid hormone, Corticotomy, Orthodontic force

## Abstract

**Background:**

Osseointegrated implants are considered as clinically non-movable. Parathyroid hormone (PTH) is known to play a significant role in the regulation of bone remodeling and in intermittent, low doses, result in osteoanabolic effects. This study aimed to investigate the effects of PTH and corticotomy, both under traction force, on osseointegrated implants.

**Methods:**

Four implants—two in each hemimandible—were placed in each of the three study mongrels. Each mongrels were designated as control, normal dose PTH (PTH-1), and high dose PTH (PTH-2) groups, with each groups further subdivided into non-surgery implant and surgery implant. After osseointegration, mechanical force with NiTi closed coil springs (500 g) was applied around each implants. Corticotomy was performed around one of four implants in each mongrels. Parathyroid hormone was administered locally on a weekly basis for 20 weeks. Clinical movement of the implants were evaluated with the superimposed 3D- scanned data, bone- microarchitectural and histologic examinations.

**Results:**

Superimposition analysis showed continuous movement of the non-surgery implant of PTH-1 group. Movement was further justified with lowest bone implant contact (adjusted BIC; 44.77%) in histomorphometric analysis. Upregulation of bone remodeling around the implant was observed in the normal dose PTH group. In the surgery implants, the remarkably higher adjusted BIC compared to the non-surgery implants indicated increased bone formation around the implant surface.

**Conclusion:**

The results indicate that the catabolic and anabolic balance of osseointegrated implants in terms of bone remodeling can be shifted via various interventions including pharmacological, surgical and mechanical force.

**Clinical relevance:**

Upregulated bone remodeling by PTH and corticotomy under continuous mechanical force showed the possible implications for the movement of osseointegrated dental implant.

## Background

The exertion of mechanical force on a tooth has been known to have direct and indirect biological effects on the surrounding tissues including the extracellular matrix, the cells of the dental pulp, periodontal ligament (PDL), alveolar bone, and the gingival soft tissue. Force induced orthodontic movement of natural teeth occurs via the bone remodeling process—bone resorption and bone formation—which occurs mainly in the PDL area [1]. However, the same cellular response cannot be expected in implants as it lacks PDL. Thus, osseointegrated dental implants, clinically considered static, may impose limitations to orthodontic treatment.

There have been reported cases of implant movement with mechanical force before and after osseointegration [2, 3]. Bone changes related to implant movement may be influenced by various factors which becomes more apparent during the treatment of ankylosed tooth or tooth movement acceleration methods. The obliteration of PDL makes ankylosed teeth rigid. In such a sense, implants are similar to ankylosed teeth in that they are immobile, thus ankylosed teeth treatment methods may be applied to study the possible movement of implants. Surgery is one treatment method for ankylosed teeth in orthodontic treatment [4]. Surgical approach such as dentoalveolar distraction osteogenesis has been attempted for canine retraction in orthodontic treatment [5]. Also, other approach for surgical intervention -corticotomy without separation of bone segment-was performed to move ankylosed tooth [6]. In this study, corticotomy and pharmacologic agent administration were applied to clarify their effects on implant movement.

Surgical approaches to in induce tooth movement ranges from comparatively simple methods such as corticotomy to rather complicated procedures such as osteotomy, a popular method used in orthodontics for rapid teeth movement [7]. The rate of teeth movement is determined by bone turnover [8], bone density [9], and PDL hyalinization [10]. Wilcko et al*.* [11] reported that active cellular response of osteoclasts and osteoblasts via multicellular mediator pathways of OPG/RANKL which is a cascade that osteoblast regulate the bone resorption and formation through secreting OPG and RANKL induced by corticotomy increases bone turnover by triggering regional acceleratory phenomenon (RAP). Corticotomy, compared to osteotomy, is comparatively less invasive, thus would be more widely used if its effects on implant movement proved significant.

Parathyroid hormone (PTH) is known to accelerate bone turnover by osteoblast mediated bone formation and osteoclast mediated bone resorption [12]. Intermittent administration of PTH influences bone formation and improves bone density [13, 14]. PTH stimulates both bone formation and bone resorption depending on its dosing regimen [15]. Further studies on the effect of PTH on implant movement under mechanical force may provide meaningful guidelines for future research.

This study aimed to examine the possibility of implant movement with PTH administration and corticotomy under mechanical force. The effects of two different dosages of PTH administration was also compared to determine the more plausible dosage.

## Methods

This experiment protocol was approved by the Ethics Committee on Animal Experimentation of the Institutional Animal Care and Use Committee, Cronex (Hwasung, South Korea) (CRONEX-IACUC 201,801,002). This study conformed the Arrive guidelines.

### Animal

The subjects of this study were three mongrels (10 months old, male, average 30 kg) and the animals were obtained through Cronex. The mongrels were selected for the study because their bone size and dentistry were able to accommodate human dental implants and allow for the use of mechanical force on implants. Orti, Bousquet [16] The subjects were housed in separate cages with regular washing system, air conditioning, and lighting. The health and oral hygiene were checked and maintained daily.

**Fig. 1:**
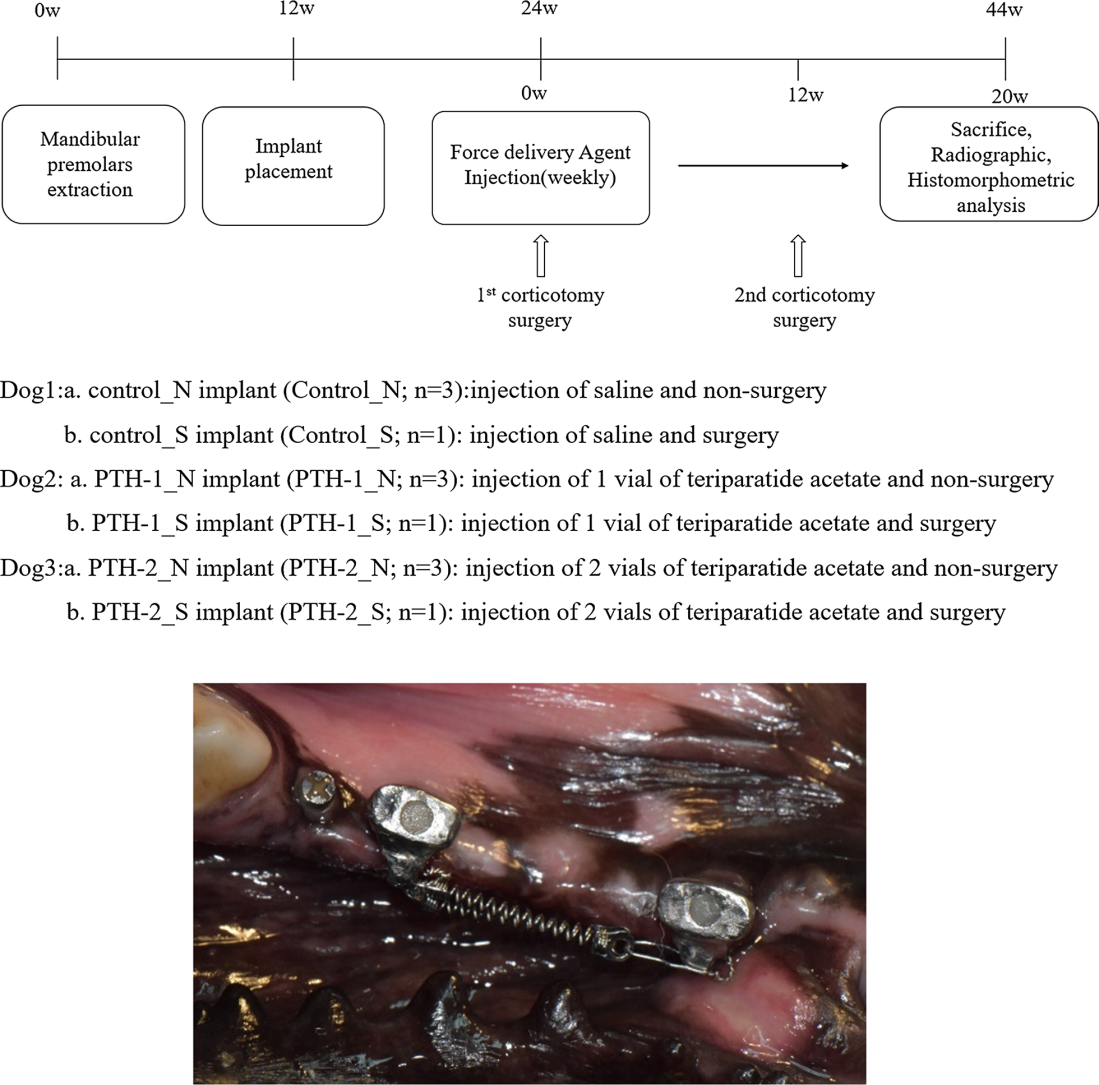
Study design and force application. **a** Study design, **b** force application; 500 g force was applied between two implants with NiTi closed coil spring. A miniscrew was placed for reference point in 3D superimposition analysis

Each mongrel underwent extraction of all mandibular premolars. After three months, four implants—two at each hemimandible, were placed in each of the mongrels. The three mongrels were each designated as control, normal dose PTH (PTH-1; TeriboneTM 56.6 μg, Asahi Kasei Corporation, Tokyo, Japan), high dose PTH (PTH-2) groups. The study groups were then further subdivided into non-surgery and surgery (corticotomy) implant. Each animal model has a total of four implants. One surgically intervened implant and three non-intervened implants (Fig. 1; Control_N, Control_S, PTH-1_N, PTH-1_S, PTH-2_N, PTH-2_S).

### Implant placement

The subjects were anesthetized with 1 mL/10 kg of intravenously administered tiletamine hydrochloride and zolazepam hydrochloride (1:1) mixture (Zoletil 50®, Virbac, Carros, France) and xylazine hydrochloride (Rumpun®, Bayer, Leverkusen, Germany). The mongrel was put to sleep via inhalation anesthesia for implant placement and antibiotics (Biotril, Komi, Siheung, South Korea) were administered for three days following surgery.

All mandibular premolars were extracted, and the implants were placed three months later. Two implants were placed in each hemimandible and they were spaced approximately 2.5 cm apart for a total of 4 implants placed in each dog. Implant TS lll SA 3.5 × 10 mm (Osstem, Seoul, South Korea) was used, and its size was selected based on mongrel bone width and nerve location [17].

Miniscrews (1.4 × 6 mm, Jeil Medical Co., Seoul, South Korea) were placed 5 mm away from the distal implant as a reference point. Osseointegration of all implants were confirmed using a radiograph of the implant and resonance frequency analyzer (Ostell Mentor, Ostell, Gothenburg, Sweden) and ISQ values of all implants were over 70. Customized abutment and metal crowns were fabricated for Nickel Titanium (NiTi) closed coil spring engagement. The maxillary first and second premolars opposite to implant placement were further extracted to avoid unnecessary occlusal loading before corticotomy surgery.

### Surgical intervention—corticotomy around implant

Corticotomy was performed twice—once before orthodontic force loading and once 12 weeks after force loading. A flap incision was made to reveal the bone, then using a piezo-ultrasonic instrument (VarioSurg 3, Nakanishi.Inc, Tokyo, Japan). Corticotomy was performed to the cancellous bone vertically in the mesial and distal position 3 mm away from the implant and horizontally 3 mm below the implant position. Only the buccal cortical bone was removed and resection was stopped when instrument reached cancellous bone. Cortical bone around the implant was additionally perforated 20 times to induce active blood flow.

Non-precious metal crowns were delivered and heavy NiTi closed coil spring (Tomy International, Tokyo, Japan) was loaded one week post first corticotomy. A force equivalent of 500 g was applied on the implants [18] (Fig. 1).

### Application of pharmacologic agents and implant movement evaluation

Pharmacologic agent was injected locally around periosteum of the implant for 20 weeks. The control group was injected with normal saline. One vial of teriparatide acetate (56.6 μg) was diluted with 1 mL saline and injected in the normal dose PTH group. Two vials of teriparatide acetate were injected in the high dose PTH group.

Radiographs and impression of the study quadrants of each mongrel were taken every two weeks to measure implant movement. The stone models at 0,1,9,12,14,16 weeks were scanned without powder, in a 3D dental scanner (Medit i500, Medit, Seoul, South Korea) and the scanned images at each time point were each superimposed with its week 0 image using the program Rapidform (Inus Technology, Seoul, South Korea). Using miniscrews as the reference point, serial superimposition of the scanned images at all different time points determined the mobility of the implants.

### Fluorescence staining for histologic analysis

New bone formation was labeled with three intramuscularly injected fluorochromes: 30 mg/kg of alizarin red (Sigma, St. Louis, USA), 10 mg/kg of calcein green (Sigma, St, Louis, USA), and 30 mg/kg of oxytetracycline yellow (Fluka, Shanghai, China) [19]. Fluorochromes were administered at 4 weeks, 6 weeks, and 55 days post second corticotomy.

### Radiographic analysis

Animals were sacrificed at 20 week after agent injection. Propofol (2-6 mg/kg) was injected and beuthanasia D solution 20 mg/kg IV was used for euthanasia. Tissue and block bones (including implants) were removed from the mandibles. The specimen were fixed in 4% paraformaldehyde (Duksan Chemicals Co. Ltd, Gyeonggi-do, Korea) for 48 h. After washing, they were assessed with micro computed tomography (μCT, SkyScan1173 Ver 1.6, Bruker-CT, Kontich, Belgium). The sample was imaged with pixel size of 29.83 μm. The voltage and current intensity of the images were 130 kV and 60μA, respectively. Eight hundred images were obtained with 500 ms exposure time, 1 mm aluminum filter, and 30 μm 2240 × 2240 pixels. Nrecon Ver 1.7.4.2 (Skysan, Aartselaar, Belgium) was used to reconstruct cross-section images from the micro CT slices.

Dataviewer and CTAn Imaging Software (Bruker micro CT, Kontich, Belgium) were used for microarchitectural analysis of the specimen. The images of each implants were cropped along the implant axis, from the area of bone-implant contact under the metal crown to 1 cm below the implant using Dataviewer. Sagittal view images were used to observe the mesial and distal areas around the implants. The regions of interest (ROIs) were determined as the 10 mm × 10 mm square area, 3 mm from the bottom of the implant. The percentage of bone volume to tissue volume (BV/TV%), trabecular pattern factor (Tb.Pf, /1 mm), trabecular number (Tb. N, /1 mm), and trabecular thickness (Tb. Th, mm) were analyzed.

### Histomorphometric analysis

Specimens were dehydrated in increasing concentration of ethanol and embedded in a mixture of ethanol and Technovit 7200 resin (Heraeus Kulzer, Wehrheimm, Germany) with an increasing ratio of resin. Following resin infiltration, the samples were hardened in an UV embedding system (KULZER EXAKT 520, Norderstedt, Germany) for a day. The undecalcified specimens were cut with an EXAKT diamond cutting system (EXAKT 300 CP, Norderstedt, Germany) and the tissue and bone were attached to a glass slide with an adhesive system. The width of the tissue section was adjusted to 40 ± 5 μm using a grinding system (EXAKT 400CS, KULZER, Norderstedt, Germany). Fluorescence images were obtained with a Pannoramic 250 Flash lll system (Histech, Budapest, Hungary). The specimens were stained with Masson Goldner Trichrome and photographed with a Pannoramic 250 Flash lll system.

Bone implant contact (BIC) was assessed with a case viewer program (3DHISTECH Ltd., Budapest, Hungary). The implant threads (a total of 12 threads) were divided into top, middle and bottom and tension and pressure side [2, 20]. The Implant surface was divided into three regions at low magnification (15× ). Tension and pressure side of implant in bone contacted surface was classified. Tension and pressure side existed in the opposite side of implant bottom because mechanical force influenced on metal crown and force was delivered to bottom of implant by center of rotation and center of resistance. BIC was calculated as the average of tension and pressure side with measurement at a higher magnification (200x). Adjusted BIC (Adj. BIC) was the average bone implant contact in tension and pressure side, except of thread exposure region.

In the fluorescence slide photographs, the outlines of the labeled bones were traced, and the distance between the inter-labeled outlines was measured. The mean distance between five points of inter-labeled outlines in 12 different regions was calculated. The mean distance between the labels was divided by the time between the injection of the labels to yield the mineral apposition rate (MAR, μm/day) [21]. The bone formation rate(BFR, μm^3^/ μm^2^/day) was determined from the formula BFR = MAR * (BS/MS) (MS; mineralizing surface, BS; bone surface). The mineralizing surface and the bone surface were calculated with Image Pro Premier (Media Cybernetics Inc., Washington Street, USA).

## Results

### Measurement of implant movement

**Fig. 2:**
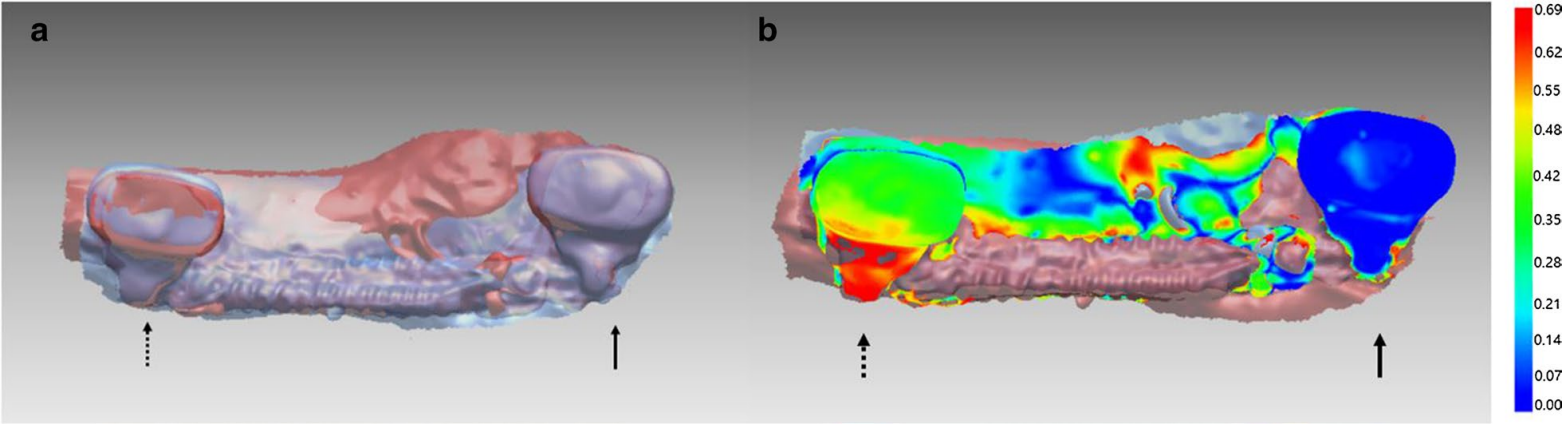
Superimposition in PTH-1_N group and PTH-1_S implant. PTH-1_N implant showed buccal movement while PTH-1_S implant showed static position. **a** Superimposition of PTH-1_N implant (dotted arrow) and PTH-1_S implant (black arrow); **b** Model deviation in superimposition of PTH-1_N implant. *blue: scanned image of the initial model (0w), red: scanned image of the final model (20w), dotted arrow: non-surgery implant, black arrow: surgery implant

The results of the 3D superimposition using the program Rapidform showed the majority of implant locations measured at final week of the study to have remained unchanged from the initial impression taken at the beginning of the study. PTH-1_N implant showed discrepancy between the initial and final implant position, indicating implant movement. PTH-1_N implant connected to PTH-1_S implant with NiTi closed coil spring also resulted in implant movement according to the superimposition analysis (Fig. 2).

### Histologic and histomorphometric analysis

**Fig. 3:**
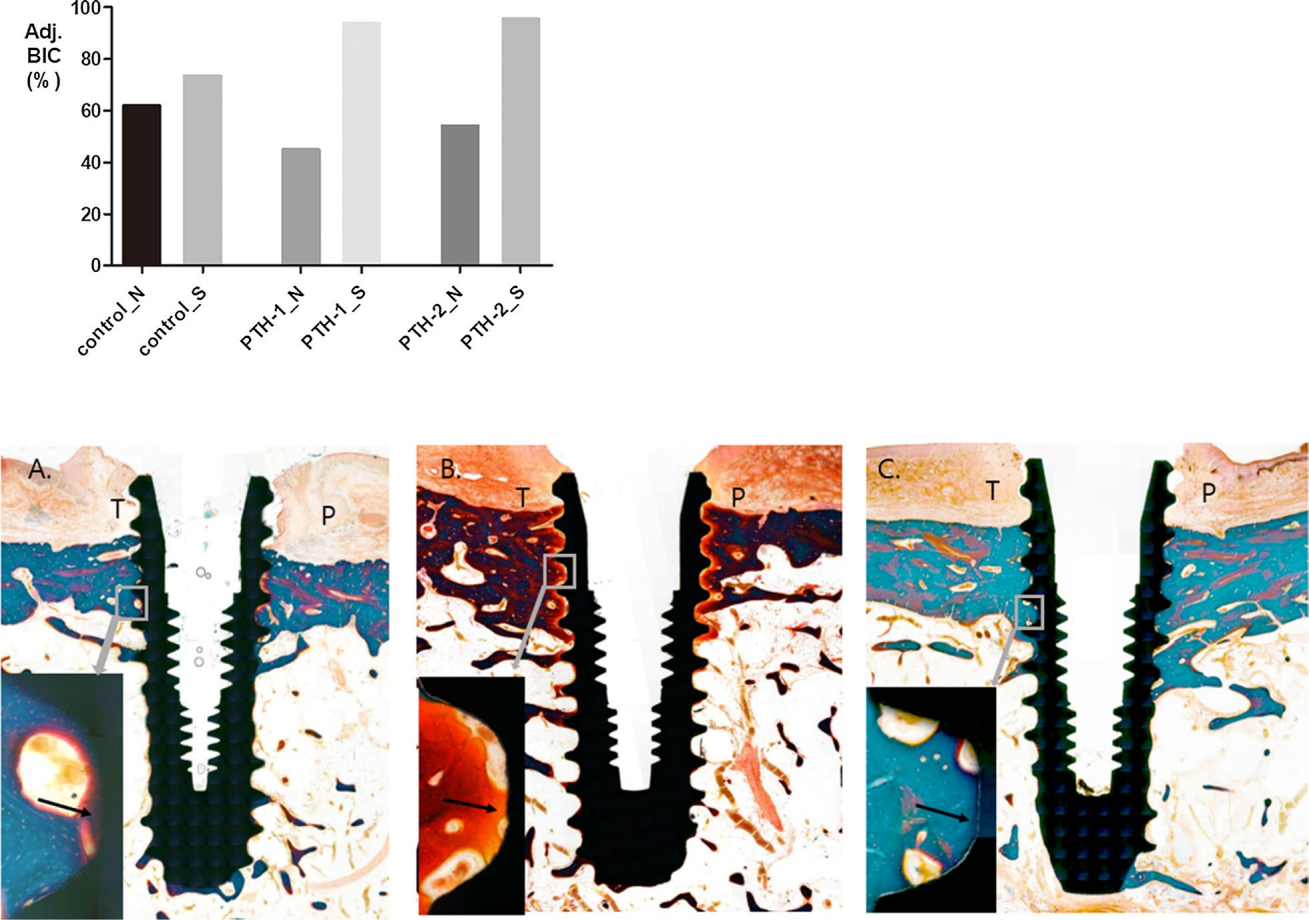
Adjusted BIC and histological section in control_N, PTH-1_N and PTH-2_N implant. Adjusted BIC (Adj.BIC) indicated mean of tension and pressure side except of thread exposure region. PTH-1_N had lowest BIC in non-surgery implants and all surgery implants indicated higher BIC. PTH-1_N implant showed bone formation (osteoid surface: rust color) and resorption (black asterisks) in implant surface that represented active bone remodeling process and least bone implant contact. (black arrow) Masson Goldner Trichrome stain. T: tension side, P: pressure side. **a** Ground section of control_N implant; **b** ground section of PTH-1_N implant; C. ground section of PTH-2_N implant. Original magnification × 10. lower left box: magnification of grey box in each implant thread surface. Original magnification × 150

The surgery implants had a remarkably higher adjusted BIC compared to those of the non-surgery implants. The adjusted BIC was lowest in the PTH-1_N implant (44.77%) and highest in the Control_N implant (61.98%) among the non-surgery groups (Fig. 3). The lowest adjusted BIC value in the PTH-1_N implant supports the result of implant movement.

As shown in Fig. 3, osteocytes and osteoid surface were found adjacent to the implant surface in the PTH-1_N implant. The BIC of the PTH-1_N implant was lowest among the three groups because many voids and osteoid surface were found in the cortical bone adjacent to the implant surface. In all surgery implants, greater bone formation around the implant was observed in the histological photographs, indicating static implant position.

**Fig. 4:**
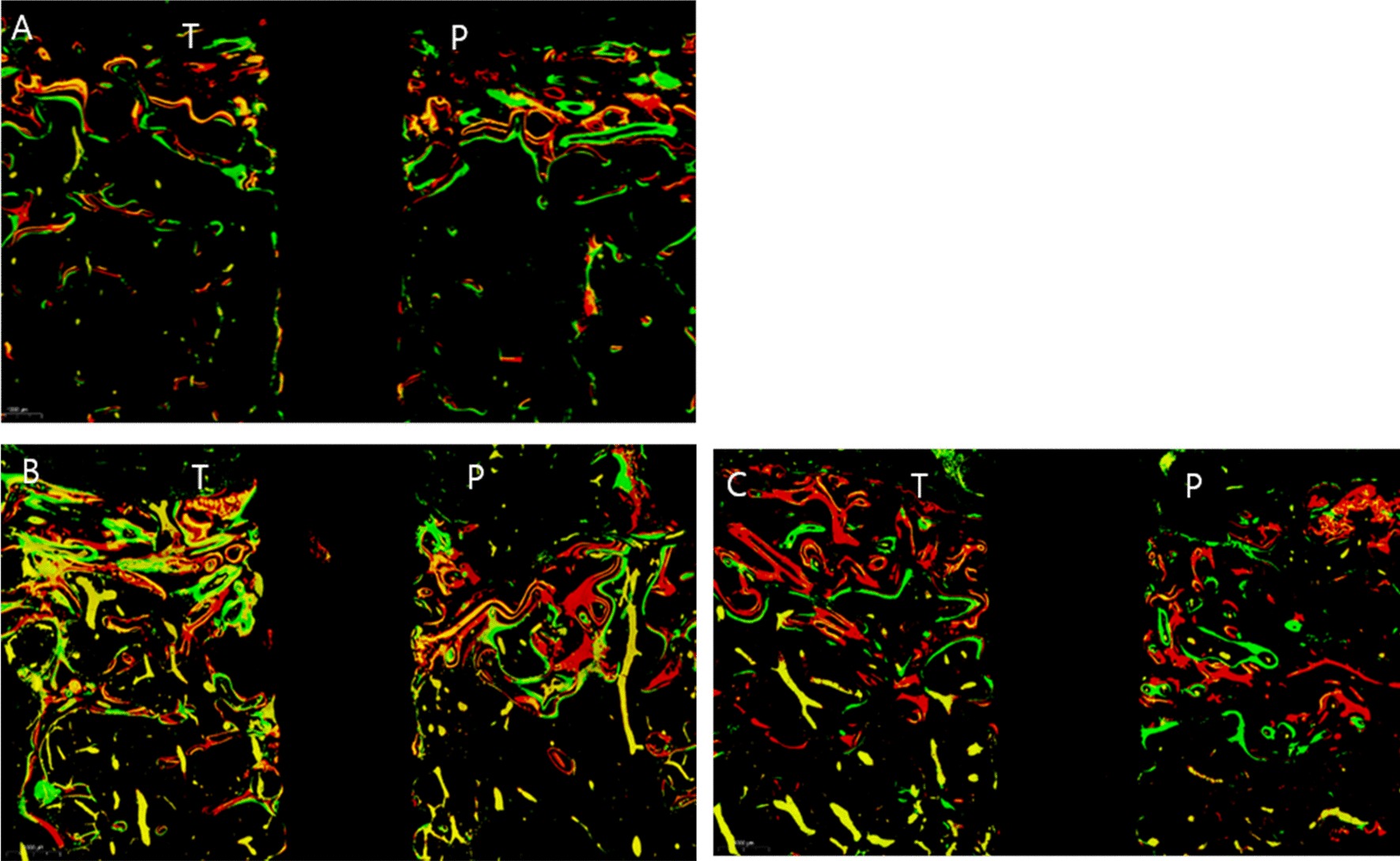
Fluorescence staining analysis of control_N, PTH-1_N, and PTH-2_N implant. PTH-1_N, PTH-2_N implant active bone formation compared to the control_N implant. PTH-1_N implant showed superior bone activation, indicating all colors and thick bone labels. Alizarin red, calcein green, and oxytetracycline yellow were injected at 4 weeks, 6 weeks, and 55 days after 2nd corticotomy, respectively. Original magnification × 10. Masson Goldner Trichrome stain. T:tension side, P:pressure side. **a** Control_N implant; **b** PTH-1_N implant; C. PTH-2_N implant

Fluorescence staining images showed greater bone activation in the PTH-1_N and PTH-2_N implants compared to the Control_N implant. In these implants, more bone labels were observed around the implants (Fig. 4). Bone remodeling was observed in all surgery implant in contact with the implant while the non-surgery implants showed bone remodeling mainly in the upper cortical bone. The PTH-1_N implant showed superior bone activation as shown by the MAR and BFR. All surgery implants showed higher MAR and BFR compared to the non-surgery implants.

Bone loss in the Control_N implant was twice that of the PTH-1_N and PTH-2_N implants. Exposure of the implant thread was higher in all surgery implants compared to the non-surgery implants (Control_N; 24.3%, PTH-1_N; 11.3%, PTH-2_N; 11.7%, Control_S; 56.3%, PTH-1_S; 46.3%, PTH-2_S; 47.9%). In the surgery implant, Control_S implant had more bone loss in the crestal bone adjacent to the implant compared to the PTH-1_S implant and PTH-2_S implant. PTH-1_N implant had the lowest thread exposure in both the non-surgery and surgery implants.

### Micro CT analysis

**Fig. 5:**
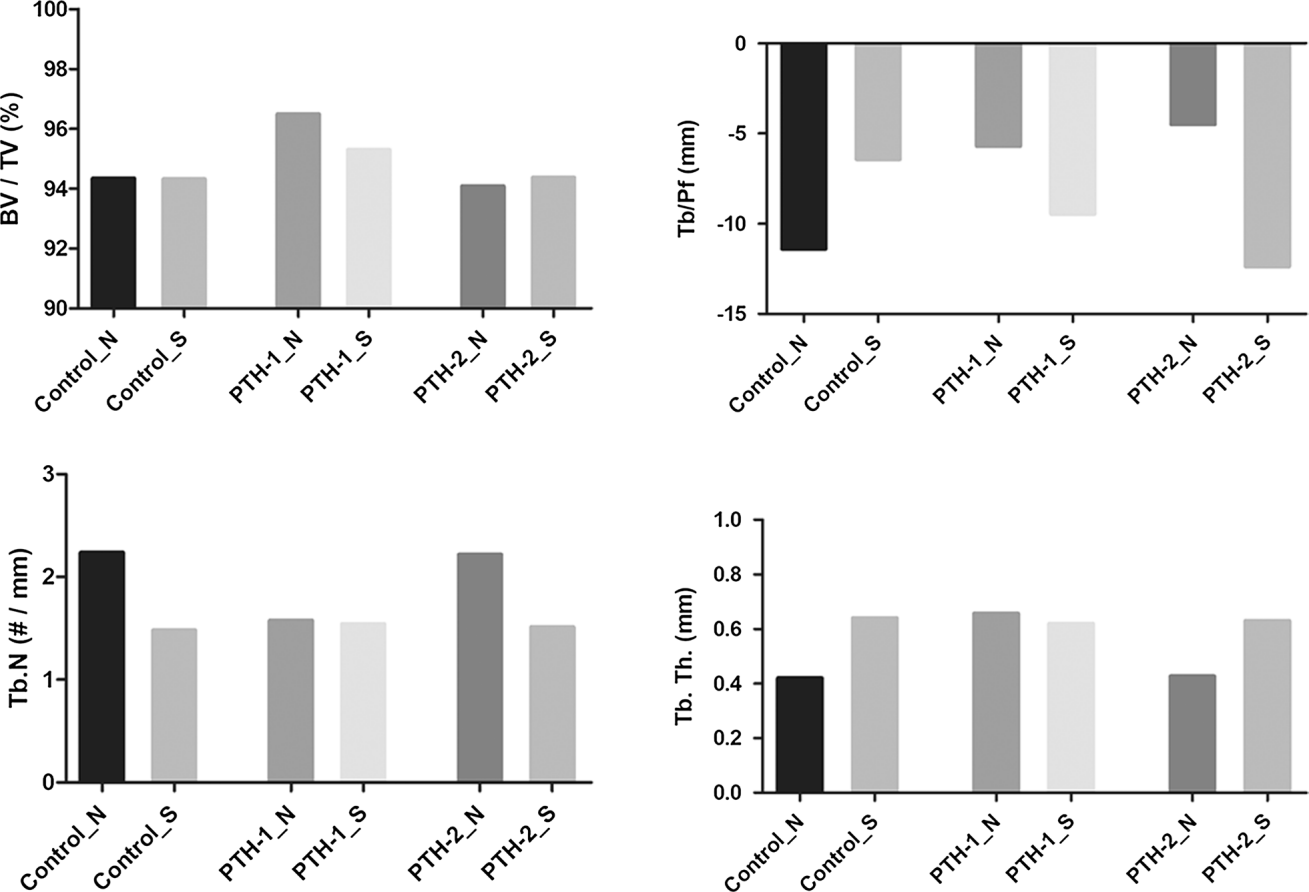
micro CT analysis. PTH-1_N showed highest BV/TV and Tb.Th among control_N, PTH-1_N, and PTH-2_N implant. Surgery implant of control, PTH-1 and PTH-2 tended to show the higher value in Tb.Th, and lower value in BV/TV and Tb.N. BV, *bone volume*; TV, *tissue volume*; Tb. N, *trabecular number*; Tb.Th, *trabecular thickness*; Tb/Pf, *trabecular pattern factor*

PTH-1_N implant showed highest BV/TV (96.5%) and Tb.Th (0.66 ± 0.19 mm) values among all non-surgery implants. As shown in Fig. 5, BV/TV, Tb. N, and Tb.Th values of PTH-2_N implant and Control_N implant did not differ significantly (PTH-2_N: 94.08%, 2.22/mm, 0.43 mm; Control_N: 94.55%, 2.29/mm, 0.41 mm) (Fig. 5).

## Discussion

Implants have long been used anchorages in orthodontics for its immobility and firmness. As a result, titanium miniscrews were commonly utilized as anchorage points for orthodontic treatments requiring teeth movement. However, Liou et al*. *[22] reported cases of non-osseointegrated orthodontic miniscrew movement upon orthodontic loading, showing that miniscrews may not necessarily remain completely stationary when acted upon force. Implant movement may be caused by factors such as low bone density or early onset of loading after implantation, both indicating a lack of osseointegration. Osseointegrated implants in low density bone may move in the first few days of orthodontic loading, but they usually settle after several weeks. This initial movement was likely due to the presence of microfractures around the implant and also to the bone remodeling and resorption process [23] rather than to conclude that they were mobile. Osseointegration in normal density bone roughly 3 months after implantation was confirmed in our experiment via radiographs and Ostell Mentor device and mobility was not exhibited during the experimental period.

3D superimposition analysis of the images at the beginning and end of the study confirmed positional change of the implant in the PTH_N implant under mechanical force. Although the stone models scanned for 3D imaging may have presented slight surface deviation errors in the mandible (less than 0.02 mm) [24], all groups were analyzed under equal conditions. Scanning of the stone model at each time was performed instead of direct intra oral scanning method to keep from uncontrolled condition such as saliva, animal’s movement and anesthetic time. In addition, we scanned the stone models because the conventional impression is accurate, similarly to the digital impression. Each implant were superimposed into 3D images using miniscrews as the reference point. Because the mongrels were still growing, miniscrew positions may have slightly been influenced by the surgical procedures, thus relative 3D superimposition images, lacking absolute fixed points, were used for analysis. To maximize the bone remodeling effect, fully grown mongrels were selected for the study. According to the histomorphometric and 3D superimposition analysis, the PTH-1_N group connected to the PTH-1_S group with a NiTi spring also showed minor positional change. Generally, a tooth loaded with a force tends to tip, translate, or move in every direction in a 3D space, depending on the point of force action and the center of resistance and rotation [25]. If the line of action passes along the center of resistance, the tooth will translate and move. The center of resistance depends on the tooth— its periodontal ligament and the cortical bone as well as the geometry of the tooth itself [26]. In this study, force was loaded at one point of the implant (buccal force) to allow for the estimation of the tipping and rotation. However, the direction of movement was difficult to conclude because the occlusal force and the external force exhibited by the daily behavior of the mongrels may have influenced implants movement.

PTH is a hormone that has known influence in bone remodeling process. Recombinant human parathyroid hormone (1–34) [teriparatide] reinforces cortical bone as well as cancellous bone [27]. Corsini et al. and Almagro et al. reported that intermittent PTH administration enhanced osseointegration and implant stability in rabbit models [28, 29]. Topical and intermittent application of PTH has also been reported to aid in alveolar bone loss recovery in rat periodontitis [30]. Thus, local and intermittent administration of PTH was performed in this study to accelerate the bone remodeling process. PTH-1 and PTH-2 groups showed more active bone remodeling compared to the control group in the micro CT analysis and histological and fluence staining finding.

PTH also has catabolic effects of bone resorption as well as anabolic effects which increases bone mass [15]. Cortical porosity and increased number of osteoclasts have been reported with low dose administration of PTH (1.25 unit/kg/day) in healthy dogs [31]. Additionally, cortical porosity involving intracortical resorption in ovariectomized rat was shown to be caused by PTH, and its effect was shown to increase with increasing dosage and frequency [32].

Movement in the normal dose PTH (PTH-1_N; 56.5 μg per week) group may have been influenced by the effect of PTH on cortical porosity. Higher BV/TV and Tb.Th values relating bone density and bone mass may also explain implant movement in the PTH-1_N group. However, the influence of higher dose of PTH (113 μg per week) on bone remodeling compared to the normal dose group was present but minimal. Schmitt et al. also reported that PTH in uremic rodent stimulated bone resorption as well as bone deposition [33] and high dose of PTH may evoke less endogenous PTH secretion by hormone homeostasis, causing less bone remodeling activity and more calcium release from the bone [34].

Corticotomy was performed on the implants to induce Regional Acceleratory Phenomenon (RAP) in tooth movement [35]. However, all the implants in the surgery groups did not move because of its significantly higher Adj. BIC despite broad thread exposure. Micro CT images presented all surgery subgroups with higher Tb. Th values, which may explain bone apposition compared to the non-surgery subgroups. However, the surgery implants showed lower BV/TV and Tb.N values which may have been due to bone loss in the upper part of the implant surface contacting the bone. In addition, bone response process in adjacent bone of implant surface after surgery could be different from that in natural teeth with PDL under traction force in that increased bone density in bone surrounding implant surface was examined in this study. Corticotomy was performed twice in this study because cellular activity due to corticotomy was reported to decrease within 11 months in humans [35]. Bone turnover rate in dogs is faster than that in humans, thus second surgery was necessary to induce cellular activity three months after the first surgery.

## Conclusion

This study demonstrated that implant movement may be feasible with intermittent PTH injection and application of mechanical force. PTH, depending on the dosage, may be applied in clinical treatments to move implants. Surgical intervention may aid in implant stability by stimulating bone formation via activated bone remodeling. There were several limitations to this study, one being the number of study participant. Increasing the study sample size may help strengthen the study validity. Additionally, the effect of surgery and the effect of PTH may not be correlated or synergic, thus future study that separately tests the independent effects of PTH and corticotomy on implant mobility is warranted. Lastly, the exact dosage of PTH beneficial for bone remodeling was understudied in this study. Thus, further studies regarding various dosage and regiment of PTH, and the amount of force induced for longer periods of time will improve the study outcomes.

## Data Availability

The data that support the findings of this study are available from the corresponding author, JW Kim, upon reasonable request.

## Abbreviations

PTH: Parathyroid hormone
BIC: Bone implant contact
PDL: Periodontal ligament
RAP: Regional acceleratory phenomenon
Adj. BIC: Adjusted bone implant contact
MAR: Mineral apposition rate
BFR: Bone formation rate
BS: Bone surface
MS: Mineralizing surface
BV: Bone volume
TV: Tissue volume
Tb. N: Trabecular number
Tb.Th: Trabecular thickness
Tb/Pf: Trabecular pattern factor

## References

[1] Krishnan V, Davidovitch Z (2009). On a path to unfolding the biological mechanisms of orthodontic tooth movement. J Dent Res.

[2] Rismanchian M (2017). Application of orthodontic immediate force on dental implants: histomorphologic and histomorphometric assessment. Ann Maxillofac Surg.

[3] Oyonarte R (2005). Peri-implant bone response to orthodontic loading: part 1. A histomorphometric study of the effects of implant surface design. Am J Orthod Dentofacial Orthop.

[4] Dolanmaz D (2010). Orthodontic treatment of an ankylosed maxillary central incisor through osteogenic distraction. Angle Orthod.

[5] Kisnisci RS (2002). Dentoalveolar distraction osteogenesis for rapid orthodontic canine retraction. J Oral Maxillofac Surg.

[6] Bousquet P (2016). Relocation of infrapositioned ankylosed teeth: description of orthodontic bone stretching and case series. J Oral Maxill Surg.

[7] Ren A (2007). Rapid orthodontic tooth movement aided by alveolar surgery in beagles. Am J Orthod Dentofacial Orthop.

[8] Verna C, Dalstra M, Melsen B (2000). The rate and the type of orthodontic tooth movement is influenced by bone turnover in a rat model. Eur J Orthod.

[9] Goldie RS, King GJ (1984). Root resorption and tooth movement in orthodontically treated, calcium-deficient, and lactating rats. Am J Orthod.

[10] Von Bohl M (2004). Focal hyalinization during experimental tooth movement in beagle dogs. Am J Orthod Dentofacial Orthop.

[11] Wilcko WM (2001). Rapid orthodontics with alveolar reshaping: two case reports of decrowding. Int J Periodontics Restorative Dent.

[12] Dempster DW (1993). Anabolic actions of parathyroid hormone on bone. Endocr Rev.

[13] Hirano T (1999). Anabolic effects of human biosynthetic parathyroid hormone fragment (1–34), LY333334, on remodeling and mechanical properties of cortical bone in rabbits. J Bone Miner Res.

[14] Hock JM (1988). Human parathyroid hormone-(l–34) increases bone mass in ovariectomized and orchidectomized rats. Endocrinology.

[15] Etoh M, Yamaguchi A (2010). Repetition of continuous PTH treatments followed by periodic withdrawals exerts anabolic effects on rat bone. J Bone Miner Metab.

[16] Orti V (2016). Benefits of mineralized bone cortical allograft for immediate implant placement in extraction sites: an in vivo study in dogs. J Periodontal Implant Sci.

[17] Taba MJ (2003). Radiographic evaluation of dental implants with different surface treatments: an experimental study in dogs. Implant Dent.

[18] Hsieh YD (2008). Evaluation on the movement of endosseous titanium implants under continuous orthodontic forces: an experimental study in the dog. Clin Oral Implants Res.

[19] Kim Y-S (2013). Effect of piezopuncture on tooth movement and bone remodeling in dogs. Am J Orthod Dentofacial Orthop.

[20] Hyzy SL (2016). Novel hydrophilic nanostructured microtexture on direct metal laser sintered Ti-6Al-4V surfaces enhances osteoblast response in vitro and osseointegration in a rabbit model. J Biomed Mater Res A.

[21] Dempster DW (2013). Standardized nomenclature, symbols, and units for bone histomorphometry: a 2012 update of the report of the ASBMR Histomorphometry Nomenclature Committee. J Bone Miner Res.

[22] Liou EJW, Pai BCJ, Lin JCY (2004). Do miniscrews remain stationary under orthodontic forces?. Am J Orthod and Dentofacial Orthop.

[23] Trisi P, Rebaudi A (2002). Progressive bone adaptation of titanium implants during and after orthodontic load in humans. Int J Periodontics Restorative Dent.

[24] Young-Hun J (2015). Three dimensional accuracy analysis of dental stone casts fabricated using irreversible hydrocolloid impressions. J Dent Rehabil Appl Sci.

[25] Smith RJ, Burstone CJ (1984). Mechanics of tooth movement. Am J Orthod and Dentofacial Orthop.

[26] Burstone CJ, Pryputniewicz RJ (1980). Holographic determination of centers of rotation produced by orthodontic forces. Am J Orthod.

[27] Jiang Y (2003). Recombinant human parathyroid hormone (1–34) [teriparatide] improves both cortical and cancellous bone structure. J Bone Miner Res.

[28] Corsini MS (2008). Effect of systemic intermittent administration of human parathyroid hormone (rhPTH[1-34]) on the resistance to reverse torque in rabbit tibiae. J Oral Implantol.

[29] Almagro MI (2013). PTH [1-34] enhances bone response around titanium implants in a rabbit model of osteoporosis. Clin Oral Implants Res.

[30] Tokunaga K (2011). Topical and intermittent application of parathyroid hormone recovers alveolar bone loss in rat experimental periodontitis. J Periodontal Res.

[31] Inoue J (1985). Bone changes with long term administration of low dose 1–34 human PTH on adult beagles. Nihon Seikeigeka Gakkai Zasshi.

[32] Takakura A (2017). Administration frequency as well as dosage of PTH are associated with development of cortical porosity in ovariectomized rats. Bone Res.

[33] Schmitt CP (2000). Intermittent administration of parathyroid hormone (1–37) improves growth and bone mineral density in uremic rats. Kidney Int.

[34] Bostrom MP (2000). Parathyroid hormone-related protein analog RS-66271 is an effective therapy for impaired bone healing in rabbits on corticosteroid therapy. Bone.

[35] Hassan AH, Al-Fraidi AA, Al-Saeed SH (2010). Corticotomy-assisted orthodontic treatment: review. Open Dent J.

